# Direct oral anticoagulants versus low molecular weight heparins for the treatment of cancer-associated thrombosis: a cost-effectiveness analysis

**DOI:** 10.1186/s12959-021-00319-1

**Published:** 2021-09-29

**Authors:** Kaidireyahan Wumaier, Wenqian Li, Naifei Chen, Jiuwei Cui

**Affiliations:** 1 Jilin University, Changchun, China; 2 Department of Cancer center, the First Hospital of Jilin University, Changchun, Jilin, 130021 China

**Keywords:** Cost-effectiveness, Direct oral anticoagulants, Low molecular weight heparin, Venous thromboembolism, Cancer-associated thrombosis

## Abstract

**Background:**

Recently, direct oral anticoagulants (DOACs) have been included in guidelines for the treatment of cancer-associated thrombosis (CAT) to be extended to suitable cancer patients. The purpose of this study was to compare the cost-effectiveness of using DOACs and low molecular weight heparins (LMWHs) for treating CAT from the perspective of the Chinese healthcare system.

**Methods:**

A Markov model was constructed to estimate the cost-effectiveness of the two strategies with a 6-month and 5-year time horizon. Input parameters were either sourced from the clinical trial, published literature. The primary outcome of the model was reported as incremental cost-effectiveness ratios (ICERs). Sensitivity analyses were performed to test model uncertainty.

**Results:**

The 6-month cost of DOACs was $ 654.65 with 0.40 quality adjusted life-years (QALYs) while the 6-month cost of LMWHs was $USD 1719.31 with 0.37 QALYs. Similarly, treatment with DOACs had a lower cost ($USD 657.85 vs. $USD 1716.56) and more health benefits (0.40 QALYs vs. 0.37 QALYs) than treatment with LMWHs in a subgroup of patients with gastrointestinal malignancy. We found treatment with DOACs would result in a large reduction in cost ($USD 1447.22 vs. $USD 3374.70) but a small reduction in QALYs (3.07 QALYs vs. 3.09 QALYs) compared with LMWHs over a 5-year time frame, resulting in an ICER of $USD 112895.50/QALYs. Sensitivity analysis confirmed the robustness of the results.

**Conclusion:**

As compared to LMWHs, DOACs can be a cost-saving anticoagulant choice for the treatment of CAT in the general oncology population and gastrointestinal malignancy population.

**Supplementary Information:**

The online version contains supplementary material available at 10.1186/s12959-021-00319-1.

## Introduction

Venous thromboembolic (VTE), which encompasses the diagnoses of both deep vein thrombosis (DVT) and pulmonary embolism (PE), is a common complication of malignancy associated with serious mortality, morbidity, and health economic consequences [1–3].

Patients with cancer are significantly more likely to develop VTE than in individuals without this disease, a ninefold increased risk is reported in such patients as compared with the normal population [4]. Of all cancer patients, while up to 50% have VTE at autopsy, with VTE being the second cause of death after cancer [5, 6]. In addition, VTE is associated with a variety of adverse consequences including an increased risk of VTE recurrence, major bleeding in cancer patients. The statistics revealed that the risk of recurrent VTE and bleeding was approximate 10–20 and 10% annually [7–9]. Moreover, VTE also has a negative impact on the quality of life of patients with malignancies [10].

Cancer-associated thrombosis (CAT) events impose a significant economic burden on the healthcare system. Compared to cancer patients without VTE, cancer patients with VTE have been shown to have three times as many all-cause hospitalizations, more days spent in the hospital, and a significantly higher number of outpatient visits [11], which results in significant healthcare costs among a cancer population of patients. Mean total hospitalization costs were 2.5-times ($17,089) higher among cancer patients with VTE compared to patients without VTE and accounted for 62% of the VTE-related total healthcare costs [12]. Nevertheless, the previous study has shown that VTE-related costs among cancer patients vary according to the type of anticoagulant treatment used [13].

Taking into account all the above, appropriate anticoagulation is of utmost importance for both clinical and economic reasons among patients with cancer. Low molecular weight heparins (LMWHs) have been recommended as the standard treatment of VTE in patients with malignancies for many years [14, 15]. Nevertheless, the implementation and adherence between recommendations and clinical behavior are suboptimal due to decreased patient satisfaction, decreased adherence rates, and increased cost of LMWHs [16]. Overcoming some of these disadvantages of LMWHs, the so-called direct oral anticoagulants (DOACs) have been recently introduced: dabigatran, rivaroxaban apixaban, and edoxaban [17–20], which represent a convenient and patient-centric anticoagulation strategy. Most notably, more recent recommendations from guidelines on the use of DOACs for the treatment of VTE to be extended to suitable cancer patients [21, 22], with emerging data supporting their safety and efficacy in the care of cancer patients [23]. However, the use of these new oral anticoagulants should be carefully considered in the decision-making process by balancing the clinical benefits and the related costs. Earlier studies found that the cost-effectiveness results for DOACs were uneven in different countries as compared with those for LMWHs [24–27], reflecting that cost-effectiveness may depend heavily on country-specific health system organizations and economics.

Given these concerns, we developed cost-effectiveness analyses on the use of the DOACs versus LMWHs in tumor patients with VTE from the Chinese healthcare system, which provides evidence of its clinical and financial benefit for decision-making.

## Materials and methods

### Overview of the model

A Markov model also called a state transition model, is a commonly used approach in decision analysis to simulate disease progression in a defined period of time. The advantage of Markov models is that Markov models can model risks over time, which enables extrapolation to the future and reduces the number of simplifying assumptions required. Markov models have been used extensively in the medical literature, and offer an appealing framework for modeling medical decision making, with potential powerful applications in decision support systems and health economics analysis. In cost-effectiveness research, Markov models are made to analyze competing treatment strategies available to a patient that can change that patient’s health state. A Markov model has a time-horizon, which is separated into fixed time periods referred to as cycles. During each of these cycles, the cohort may transition between a finite number of health states according to appropriate probabilities. Costs and effects are typically incorporated into these models as a mean value per state per cycle. It is thus possible to calculate the expected cost and expected outcome of each option under evaluation. For a given option, the expected cost (outcome) is the sum of the costs (outcomes) of each consequence weighted by the probability of that consequence.

We constructed a Markov model using proprietary software (TreeAge Pro 2011 Software, Williamstown, MA) concerning a hypothetical reference case, which was similar to the approach adopted in previously published studies [24, 26–29]. A hypothetical cancer population of 64-year-old, 70 kg, and with VTE event receiving treatment with DOACs or LMWHs was considered for the model. A 1-month cycle length with a 6-month and 5-year time horizon was used. The 6-month time horizon was chosen based on the applied data period from the randomized controlled trials (RCTs) and the 5-year time horizon was chosen as it is commonly used to reflect important clinical and economic impacts of DOACs for CAT and general cancer survival [26, 30–32].

**Fig. 1:**
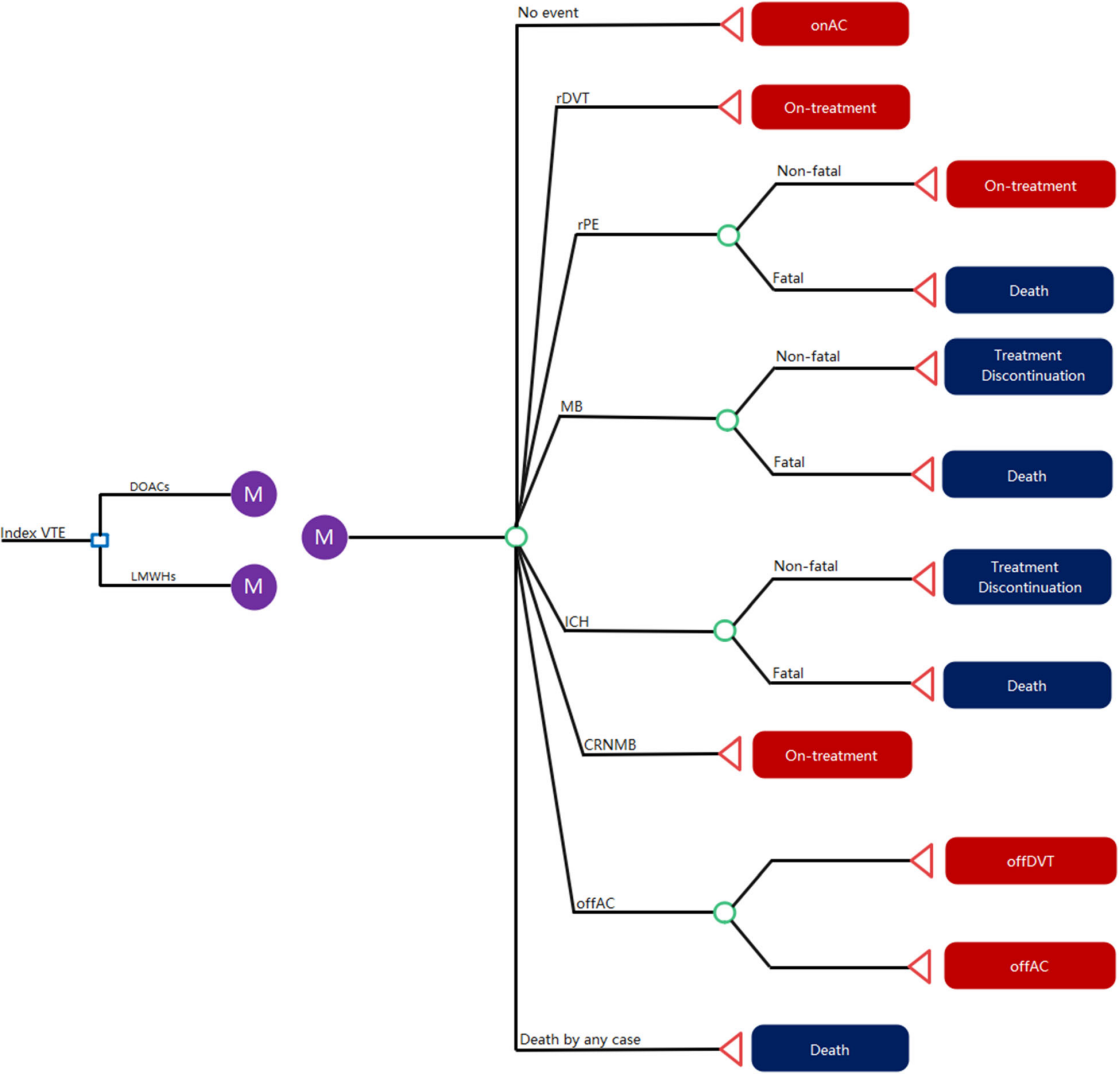
Model diagram. Abbreviations: DOACs, direct oral anticoagulants; LMWHs, low molecular weight heparins; onAC, on anticoagulation treatment with no event; rDVT, recurrent deep-vein thrombosis; rPE, recurrent pulmonary embolism; ICH, intracranial hemorrhage; CRNMB, clinically relevant non-major bleeding; offAC, anticoagulant treatment discontinuation; offDVT, risk of deep-vein thrombosis while off-treatment; offPE: risk of pulmonary embolism while off-treatment

The Markov model, consisting of 10 health states, as depicted in Fig. 1, included on anticoagulant treatment, off anticoagulant treatment, recurrent pulmonary embolism (rPE), recurrent deep vein thrombosis (rDVT), intracranial hemorrhage (ICH), non-ICH major bleeding (MB), clinically relevant non-major bleeding (CRNMB), PE-related death, MB-related death, and death by any case. Specifically, patients entered the model following a VTE event, at the beginning of the decision tree, the patients would receive one of the four following agents for the treatment of VTE in cancer patients: apixaban, rivaroxaban, edoxaban, or LMWHs that dosages for agents were based on their respective trials, and then they either remained in their current on-treatment state, moved to an event state, transitioned to an off-treatment state or died, during the course of 1-monthly cycles. Each state was associated with a cost and utility weighting to calculate the total costs and quality adjusted life-years (QALYs) of patients simulated in the model.

### Parameters of the model input

The clinical inputs for various event probabilities used in the model are summarized in Table 1. The probabilities of events of DOACs and LMWHs during the first 6 months were obtained from 4 good quality RCTs including the Hokusai VTE Cancer trial [33], Select-D [34], the Caravaggio study [35], and ADAM VTE trial [36] in which each of the DOACs were directly compared with LMWHs for the treatment of VTE focused on patients with active cancer. All studies had the primary efficacy outcome (recurrent VTE) and the primary safety outcome (major bleeding). In each study, patients were followed for at least 6 months. DOACs were shown to be noninferior to dalteparin for recurrent VTE and major bleeding. Bleeding was more common in patients with GI malignancies taking edoxaban and rivaroxaban compared with dalteparin [33, 34]. In contrast, apixaban was not associated with an increased risk of bleeding compared with dalteparin in the ADAM and Caravaggio trials [35, 36]. Transition probabilities (TP) for 7–12 months were derived directly from the Hokusai-VTE study and the same estimates were extrapolated to the time horizon beyond 12 months (Supplemental Table 1). The time-varying TP for recurrent VTE when off anticoagulant treatment was estimated from a large population study of cancer patients [37]. The probability of events of bleeding seen in patients with gastrointestinal malignancy was derived from the randomized controlled trials as above, including the Hokusai-VTE [33], Select-D [34], Caravaggio [35] trials. The event rates were translated into monthly transition probabilities with the following formula: Tp = 1 − (1 − p)^(1/n) (with Tp = monthly probability of events, *p* = event probability as reported in the literature, and *n* = number of months).

**Table 1:** Parameters for model input with a cycle length of 1 month

Parameters	Base case	Ranges	Distribution	Source
1-6 month transition probabilities (tp) ^a^, %				
DOACs				
Recurrent DVT	0.33	0.26–0.40	β	[33–36]
Recurrent PE	0.54	0.43–0.65	β	[33–36]
Fatal PE	4.45	3.56–5.34	β	[33–36]
MB	0.70	0.56–0.84	β	[33–36]
Fatal MB	0.28	0.22–0.34	β	[33–36]
ICH	0.02	0.016–0.024	β	[33–36]
Fatal ICH	0	0	β	[33–36]
Major GI bleeding	1.17	0.94–1.40	β	[33–36]
CRNMB	1.81	1.45–2.17	β	[33–36]
Death of any case	4.46	3.57–5.35	β	[33–36]
Treatment discontinuation	2.91	2.33–3.49	β	[33–36]
LMWHs				
Recurrent DVT	0.61	0.49–0.73	β	[33–36]
Recurrent PE	0.74	0.59–0.89	β	[33–36]
Fatal PE	2.24	1.79–2.69	β	[33–36]
MB	0.47	0.38–0.56	β	[33–36]
Fatal MB	1.26	1.01–1.51	β	[33–36]
ICH	0.08	0.06–0.10	β	[33–36]
Fatal ICH	5.45	4.36–6.54	β	[33–36]
Major GI bleeding	0.56	0.45–0.67	β	[33–36]
CRNMB	1.09	0.87–1.31	β	[33–36]
Death of any case	4.52	3.62–5.42	β	[33–36]
Treatment discontinuation	4.73	3.78–5.68	β	[33–36]
OffDVT	1.90	1.52–2.28	β	[37]
OffPE	2.03	1.63–2.44	β	[37]
Costs, $				
DOACs 1st month	207.34	165.87–248.81	γ	[38]
DOACs 2nd month onwards	825.89	660.71–991.07	γ	[38]
LMWHs (enoxaparin) 1st month	149.91	119.93–179.89	γ	[38]
LMWH (enoxaparin)2nd month onwards	412.94	365.30–547.94	γ	[38]
DVT	693	329–941	γ	[39]
PE	1121	448–1793	γ	[39]
MB	654	603–704	γ	[40]
ICH	3834	2684–4984	γ	[41]
CRNMB	8.25	5.77–10.72	γ	[41]
Utilities			β	
Base	0.95	0.76–1.00	β	[42]
DOACs	0.95	0.76–1.00	β	Assumed^b^
LMWHs (enoxaparin)^a^	0.85	0.68–1.00	β	[42]
DVT	0.84	0.67–1.00	β	[43]
PE	0.63	0.50–0.76	β	[43]
MB	0.65	0.52–0.78	β	[43]
ICH	0.33	0.26–0.40	β	[43]
CRNMB^a^	0.91	0.73–1.00	β	[44]

*DVT* Deep vein thrombosis, *PE* Pulmonary embolism, *MB* Major bleeding, *ICH* Intracranial hemorrhage, *CRNMB* Clinically relevant non-major bleeding, *DOACs* Direct oral anticoagulants, *LMWHs* Low molecular weight heparins, *offDVT* Risk of deep-vein thrombosis while off-treatment, *offPE* Risk of pulmonary embolism while off-treatment

^a^Upper and lower bounds estimated to vary ±20% of the mean value for these input parameters estimates

^b^Utility associated with DOACs treatment was assumed to be same as base

The cost analysis was evaluated from the healthcare system perspective setting in China. In analysis, it includes patients’ direct medical costs related to drugs and complications, without considering indirect costs and direct non-medical costs. The daily drug acquisition costs of DOACs and LMWHs were collected from public databases [38]. An average of the edoxaban, rivaroxaban, and apixaban total drug costs was used for the DOACs arm. The wording DOACs refer to apixaban, edoxaban, or rivaroxaban, where did not include dabigatran as dabigatran was not used in any study. Costs for enoxaparin were used for this model due to its widespread use in China, although the clinical trials in cancer patients have used LMWHs such as dalteparin. Monthly costs (each cycle) were derived from 30-day prescriptions of the drugs at the labeled dosing frequency. The cost of symptomatic DVT or PE considered both the diagnosis and hospitalization costs incurred for such events [39]. The resource use in managing a major bleeding event was based on a Chinese study analyzing the costs for inpatient admissions due to major bleeding events [40]. Costs for other states were also based on values in the previously published literature. All the costs were calculated and reported in US dollars (USD) with the average exchange rate in 2020 (¥ = $0.14). Also, a discount rate of 5% was used, as recommended by Chinese guidelines for pharmacoeconomic evaluations [45] each year. All costs are reported in Table 1.

The quality adjusted life-years (QALYs; duration times utility) was incorporated in the model by using the values of utility. Evidence from previously published literature was used to determine the various utility values. As the literature on the utility of various events in cancer patients with VTE events is scarce, thus, most data were obtained from VTE patients without cancer [42, 46]. The base utility was considered to be 0. 95 and oral anticoagulant treatments were assumed not to change the utility value [42]. The utility inputs of the direct effects of the treatment drugs and the series of clinical events are reported in Table 1.

### Analysis

We assessed the cost-effectiveness of treatment with DOACs compared to LMWHs among patients with CAT. In addition, given the increased rate of bleeding seen in patients with gastrointestinal malignancy on edoxaban [33] and rivaroxaban [47], a subgroup cost-effectiveness analysis was performed on this patient population.

The primary outcome measure of this study is the incremental cost-effectiveness ratio (ICER), which is the ratio of incremental cost and incremental effect between the two groups. According to the world health organization (WHO) recommendation, When the ICER was less than three times the gross domestic product (GDP) per capita, cost-effectiveness would be considered [48]. We used three times the per-capita GDP of China in 2020 ($10,142.58) with willingness to pay (WTP) thresholds of US $30,427.74 per QALY as the cost-effectiveness threshold.

To explore the effect of parameter uncertainty in the model, we performed one-way sensitivity analysis (OWSA) and probabilistic sensitivity analysis (PSA). In OWSA, the value range of each parameter was either based on the reported or estimated 95% CIs in the referenced studies or determined by assuming a 20% change from the point estimate in the base-case analysis. The 10 most influencing parameters were presented in a tornado diagram. PSA was performed using a Monte Carlo simulation with 1000 iterations. The distributions assumed for the input parameters were gamma (cost), beta (utility weights and TP), and log-normal (RR). All the analyses were performed in TreeAge Pro 2011.

## Results

### Main results

The results of the cost-effectiveness analysis are summarized in Table 2. During the first 6 months, the cost of the DOACs treatment was $ 654. 65 and $ 1719.31 for the LMWHs treatment. The effectiveness of the DOACs treatment was 0. 40 QALYs; for the LMWHs treatment it was 0. 37 QALYs. The estimated ICER was $ 32,922.16 and in favor of DOACs. In the outcome analysis of the 60-month time frame, the cost of the DOACs strategy was $ 1447. 22 and $ 3374. 70 with LMWHs. The QALYs associated with DOACs was 3. 07; for the LMWHs it was 3. 09 QALYs. The ICER of DOACs compared to LMWHs was $ 112,895.50 per QALY. In the subgroup analysis of those patients with gastrointestinal malignancy, the results showed that DOACs were the preferred strategy over LMWHs with low cost ($ 657.85 vs. $1716.56) and high QALYs (0.40 QALYs vs. 0.37 QALYs).

**Table 2:** Cost-effectiveness analyses

	Costs	ΔCosts	QALYs	ΔQALYs	ICER
6-month time horizon					
DOACs	654.65	− 1064.66	0.40	0.03	−32,922.16
LMWHs	1719.31		0.37		
5-year time horizon					
DOACs	1447.22	− 1927.48	3.07	−0.02	112,895.50
LMWHs	3374.70		3.09		
Subgroup	0.28				
DOACs	657.85	− 1058.71	0.40	0.03	−32,821.83
LMWHs	1716.56		0.37		

*DOACs* Direct oral anticoagulants, *LMWHs* Low molecular weight heparins, *ICER* Incremental cost-effectiveness ratio, *QALYs* Quality adjusted life-years

### Sensitivity analysis

**Fig. 2:**
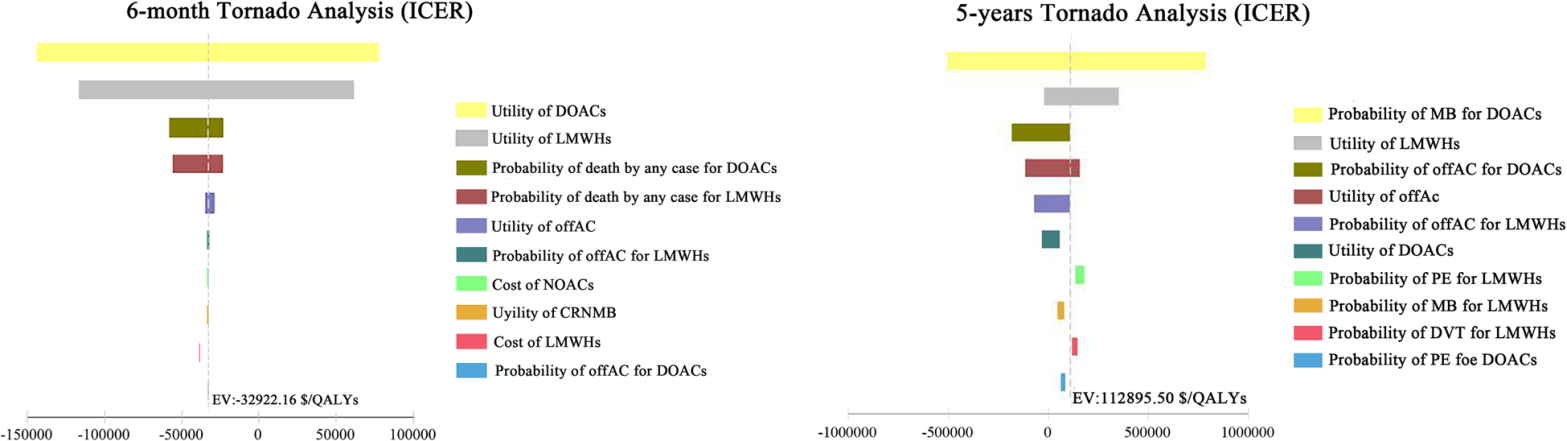
One-way sensitivity analyses (Tornado diagram) over 6-month (left) and 5-year time horizon (right). In the graph, a horizontal bar is generated for each variable being analyzed. Incremental cost is displayed on the horizontal axis, so each bar represents the selected node’s range of incremental values generated by varying the related variable. A wide bar indicates that the associated variable has a large potential effect on the expected value of the model. This variable with the widest bar (potentially the most critical uncertainty) is plotted on the top. DOACs, direct oral anticoagulants; LMWHs, low molecular weight heparins. CRNMB: clinically relevant non-major bleeding; offAC, anticoagulant treatment discontinuation; ICER, incremental cost-effectiveness ratio; QALYs: quality adjusted life-years (QALYs)

**Fig. 3:**
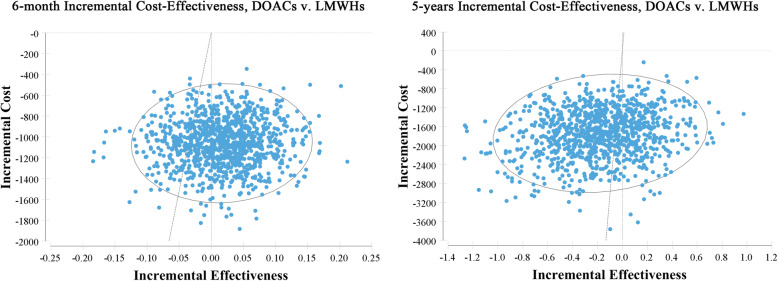
Probabilistic sensitivity analysis of the cost-effectiveness using Monte Carlo simulation over 6-month (left) and 5-year time horizon (right). The vertical axis represents incremental cost in USD, horizontal axis represents incremental effectiveness in QALYs, blue spots represents 1000 draws of the probabilistic analysis and the the slope of a line intersecting the origin of the plot represents the willingness to pay (WTP) limit. Values on the right side of the WTP line are considered cost-effective

In the sensitivity analysis, we assessed the robustness of the model over a 6- month time horizon and a 5-year time horizon. Tornado diagrams illustrating the 10 most influential variables in descending order of influence are depicted in Fig. 2. According to the OWSA, the 6-month analysis showed that the most sensitive parameters included the utilities of DOACs and LMWHs. The ICER was particularly sensitive to non-ICH major bleeding (MB) events in treatment with DOACs compared to Enoxaparin at five years intervals, respectively. Considering the estimated results for PSA, the majority of simulations showed that the treatment with DOACs was more cost-effective than the equivalent duration of LMWHs treatment. Overall, these analyses suggest that the model outcomes were robust. The scatterplot of these repetitions is shown in Fig. 3.

## Discussion

To our knowledge, this is the first study to evaluate the cost-effectiveness of all available DOACs simultaneously compared with LMWHs for treatment in patients with cancer-associated VTE. In the present study, an economic analysis of four potentially competing treatment agents in different treatment durations (5-year and 6-month time horizon, respectively) was undertaken from a Chinese healthcare payer perspective, including three new oral anticoagulants (edoxaban, rivaroxaban, and apixaban) and one low molecular weight heparin (enoxaparin). We conclude that, in Chinese, DOACs can be a reasonable anticoagulant choice for many patients with cancer-associated VTE.

In our cost-effectiveness analysis of different DOACs vs LMWHs for the treatment of CAT over a 6-month time frame, our results showed that DOACs are cost-effective, which also has been found in subgroup analysis. DOACs remained more effective and less costly than LMWHs under most of the scenarios explored by sensitivity analysis. The one-way sensitivity analysis revealed that the utility of DOACs and LMWHs had the greatest impact on the results. The explanation for the differences seen in the sensitivity analysis is the higher cost of new oral anticoagulants and lower costs of enoxaparin and managing VTE events in China in comparison with those in developed countries [49–55], lead the strategies will approach equipoise in which case differences in patient preference between injection and oral therapy will become the major determinant between strategies. The PSA demonstrated the robustness of the results, as most of the points in the PSA scatter plot were located in the upper right zone. An economic comparison of edoxaban and LMWHs in the US showed a lower cost of treatment with rivaroxaban ($ 6061 vs $19398) as well as similar QALYs gained (0. 34 QALYs vs 0. 35 QALYs) for a 6- month time horizon [27]. Other studies for a 6-month time horizon [25], from the Netherlands, found that rivaroxaban was the most cost-effective treatment choice compared with LMWHs.

Our analysis suggests that DOACs is a cost-saving treatment option with only a modest decrease in QALYs as compared to LMWHs over 5 years. The ICER for DOACs vs. LMWHs was $112,895.50, which is far higher than the threshold of US $30,427.74 (three times GDP per capita of China in 2020).

Additionally, this uncertainty and variation surrounding the model inputs were evaluated in our sensitivity analysis and demonstrated that despite these uncertainties most of the conclusions remained the same. OWSA indicated that the major bleeding in treatment with DOACs had a high influence on ICER. The potential reason for this is that the more frequent events of MB with DOACs compared with LMWHs. MB events are very burdensome and frequently severely disabling, leading to low QALYs despite it is modest decrease. Recent studies found that DOACs may be associated with low MB events for the treatment of cancer-associated VTE in Asian patients than in non-Asian patients [56]. Furthermore, a significant decrease in GI bleeding risk was observed with DOACs [56]. So DOACs can be a more cost-effective treatment compared to LMWHs in Chinese patients with CAT. Further prospective studies are needed to confirm these findings. Moreover, the favorable pharmacoeconomic profile was robust in the probabilistic sensitivity analysis.

Several cost-effectiveness analyses have been conducted in different countries [24, 25], but none of them evaluated all DOACs simultaneously, with majority of them focusing on single DOACs only. However, only one study from a US payer perspective was found that compared the cost-effectiveness of different DOACs (Edoxaban+Rivaroxaban) compared with LMWHs [26], have demonstrated DOACs were cost-saving options. The other previous study in the Brazilian population showed that edoxaban is a cost-saving alternative to LMWH for the management of CAT with incremental cost and QALYs increases were $ 16,654.27 and 3.2, respectively [24].

Not surprisingly, as with all cost-effectiveness analyses, there are some uncertainties and limitations associated with our analysis. Firstly, given the absence of local data, clinical and safety estimates were derived from different randomized controlled treatment results in multiple countries rather than the Chinese or the Asian population specifically, and they may not reflect real-world observations. Secondly, data of utilities that are specially aimed at patients with cancer remain scarce. we extrapolated the most utility values from the general medical patients to cancer patients with VTE, which may be overestimated. Future studies are needed to directly assess the utility of cancer patients. Whereas, the sensitivity analyses suggest that the results were robust and unlikely to be significantly affected by variations in utility variables. Thirdly, Not all relevant costs were included, the current analysis only included direct medical costs, without considering information about indirect and direct non-medical costs, which may underestimate the total treatment cost per patient. Finally, The model uses a Chinese societal perspective, however, the costs of both DOACs and LMWHs will vary depending on which country or specific health system is evaluating the use of these agents, and this could affect the transfer of cost-effectiveness results from one country to another in a healthcare context.

## Conclusion

In conclusion, this economic evaluation has shown that DOACs were estimated to be a cost-saving option when compared to LMWHs for the treatment of CAT in Chinese patients, both the 60-month (extrapolated) and the 6-month (data-driven) horizons. We believe the results of this study would be an important addition to inform the limited data about the economic impact of VTE among cancer patients.

## Supplementary Information

**Additional file 1.** .

## Data Availability

All data generated or analysed during this study are included in this published article and its supplementary information files.

## Abbreviations

DOACs: Direct oral anticoagulants
CAT: Cancer-associated thrombosis
LMWHs: Low molecular weight heparins
ICERs: Incremental cost-effectiveness ratios
VTE: Venous thromboembolic
DVT: Deep vein thrombosis
PE: Pulmonary embolism
RCTs: Randomized controlled trials
rPE: Recurrent pulmonary embolism
rDVT: Recurrent deep vein thrombosis
ICH: Intracranial hemorrhage
MB: Non-ICH major bleeding
CRNMB: Clinically relevant non-major bleeding
TP: Transition probabilities
USD: US dollars
offAC: Anticoagulant treatment discontinuation
offDVT: Risk of deep-vein thrombosis while off-treatment
offPE: Risk of pulmonary embolism while off-treatment
WTP: Willingness to pay
OWSA: One-way sensitivity analysis
PSA: Probabilistic sensitivity analysis
QALYs: Quality adjusted life-years
GDP: Gross domestic product
WHO: World health organization
