# Supplementary material for: Direct oral anticoagulants versus low molecular weight heparins for the treatment of cancer-associated thrombosis: a cost-effectiveness analysis
Source: Thromb J. 2021 Sep 29;19:68. doi: 10.1186/s12959-021-00319-1 (PMC8479897; doi:10.1186/s12959-021-00319-1)
Supplement: Supplementary file 1 — Additional file 1. . [file 12959_2021_319_MOESM1_ESM.doc]

**Authors name list:** Kaidireyahan.Wumaier, Wenqian Li, Naifei Chen, Jiuwei Cui.

Supplemental Table 1. 7-12month transition probabilities (tp) for with a cycle length of 1 conth

| Parameters | Base case | | Ranges | Distribution | | Source |
| --- | --- | --- | --- | --- | --- | --- |
| 7-12month transition probabilities (tp) [^a^](#a3), % | |  |  |  |  | |
| DOACs |  | |  |  |  | |
| Recurrent DVT | 0.13 | | 0.10-0.16 | β |  | |
| Recurrent PE | 0.06 | | 0.05-0.07 | β |  | |
| Fatal PE | 4.45 | | 3.56-5.34 | β |  | |
| MB | 0.11 | | 0.09-0.13 | β |  | |
| Fatal MB | 0.28 | | 0.33-0.34 | β |  | |
| ICH | 0 | | 0 | β |  | |
| Fatal ICH | 0 | | 0 | β |  | |
| CRNMB | 0.19 | | 0.15-0.23 | β |  | |
| Death of any case | 1.12 | | 0.90-1.34 | β |  | |
| Treatment discontinuation | 2.91 | | 2.33-3.49 | β |  | |
| LMWHs |  | |  |  |  | |
| Recurrent DVT | 0.21 | | 0.17-0.25 | β |  | |
| Recurrent PE | 0.06 | | 0.05-0.07 | β |  | |
| Fatal PE | 2.24 | | 1.79-2.24 | β |  | |
| MB | 0.06 | | 0.05-0.07 | β |  | |
| Fatal MB | 1.26 | | 1.79-2.69 | β |  | |
| ICH | 0 | | 0 | β |  | |
| Fatal ICH | 0 | | 0 | β |  | |
| CRNMB | 0.24 | | 0.19-0.29 | β |  | |
| Death of any case | 1.10 | | 0.88-1.32 | β |  | |
| Treatment discontinuation | 4.73 | | 3.78-5.68 | β |  | |
| Off DVT | 0.73 | | 0.58-0.88 | β |  | |
| Off PE | 0.33 | | 0.26-0.40 | β |  | |

DVT, deep vein thrombosis; PE, pulmonary embolism; MB, major bleeding; ICH, intracranial hemorrhage; CRNMB, clinically relevant non-major bleeding; DOACs, direct oral anticoagulants; LMWHs, low mole-cular-weight heparins; offDVT, risk of deep-vein thrombosis while off-treatment; offPE: risk of pulmonary embolism while off-treatment.

^a^: Upper and lower bounds estimated to vary ± 20% of the mean value for these input parameters estimates.
